# Complete mitogenomes of two species of *Cephenemyia* and *Pharyngomyia picta*, and a comparison with other Oestrinae

**DOI:** 10.1007/s00436-025-08623-9

**Published:** 2026-01-13

**Authors:** Gaël Aleix-Mata, Pablo Mora, Paloma Prieto-Yerro, Roser Velarde, Alexander P. Saveljev, Antonio Sánchez-Camacho, Einer F. Mendoza, Eugenia E. Montiel, Jesús M. Pérez, Antonio Sánchez

**Affiliations:** 1 Cos de Banders, Govern d’Andorra, Av. De la Bartra AD200, Encamp, Andorra; 2 Departamento de Biología Experimental, Área de Genética, Universidad de Jaén, Campus de las Lagunillas s/n, Jaén, 23071 Spain; 3 Parque Natural Sierras de Cazorla, Segura y Las Villas, C/Martínez Falero 11, Cazorla, Jaén, 23470 Spain; 4 Wildlife Ecology & Health Group (WE&H) and Servei d’Ecopatologia de Fauna Salvatge (SEFaS), Departament de Medicina i Cirurgia Animals, Facultat de Veterinària, Universitat Autònoma de Barcelona, Bellaterra, 08193 Spain; 5 Department of Animal Ecology, Russian Research Institute of Game Management and Fur Farming, Kirov, Russia; 6 Departamento de Genética, Facultad de Ciencias, Universidad de Granada, Granada, 18071 Spain; 7 Departamento de Biología Animal, Biología Vegetal y Ecología, Universidad de Jaén, Campus Las Lagunillas, s.n., Jaén, 23071 Spain

**Keywords:** Cephenemyia auribarbis, Cephenemyia ulrichii, Pharyngomyia picta, Mitogenome, Mitochondrial genome, Oestrinae

## Abstract

This study reports for the first time the complete sequencing of the mitogenome of three Oestridae species: *Cephenemyia auribarbis*, *C. ulrichii*, and *Pharyngomyia picta* (Diptera: Oestridae). These three mitogenomes were assembled and annotated from specimens collected in southern and northern Spain (*C. auribarbis* and *P. picta*, respectively) and in Russia (*C. ulrichii*). They were then compared with the mitogenomes of 18 other Oestridae species available in GenBank. All three analyzed mitogenomes exhibited a conserved gene content and structure comprising 13 protein-coding genes, 22 tRNAs, two rRNAs, and a control region, with a high A + T content characteristic of the Oestridae. Intraspecific variability in length and sequence was detected in *C. ulrichii* and *P. picta*. Comparative analyses revealed conserved start/stop codons and consistent PCGs, tRNA, and rRNA gene organization. The D-loop region had the highest sequence variability. The phylogenetic analyses of 24 complete mitogenomes confirmed the monophyly of the genus *Cephenemyia*, with *Pharyngomyia* as its closest relative. Together, these genera formed a well-supported clade with *Oestrus* and *Rhinoestrus*. These results served to reinforce previously postulated phylogenetic hypotheses and underscored the utility of mitogenomic data in resolving the evolutionary relationships within the Oestridae. The mitogenomes described in this study offer significant molecular resources for future research into the taxonomy, phylogeny, and molecular evolution of bot flies.

**Supplementary Information:**

The online version contains supplementary material available at 10.1007/s00436-025-08623-9.

## Introduction

Oestrids (Diptera: Oestridae Leach, 1815) are obligate parasites of mammals during their larval stage. The females of nasal/pharyngeal bots (subfamily Oestrinae) are larviparous and deposit or eject packets of larvae into the nostrils of their hosts. The larvae move into the nasal, pharyngeal, and frontal cavities of the host, where they feed on cellular debris and mucosal secretions until maturity (Colwell 2001). The family Oestridae includes four subfamilies: Cuterebrinae, Gasterophilinae, Hypodermatinae, and Oestrinae (Pape 2001; Scholl et al. 2019), and the subfamily Oestrinae includes nine genera: *Cephenemyia* (Latreille, 1818), *Cephalopina* (Strand, 1928), *Gedoelstia* (Rodhain and Bequaert, 1913), *Kirkioestrus* (Rodhain and Bequaert, 1915), *Oestrus* (Linnaeus, 1758), *Pharyngobolus* (Brauer, 1866), *Pharyngomyia* (Schiner, 1861), *Rhinoestrus* (Brauer, 1886), and *Tracheomyia* (Townsend, 1916) (Angulo-Valadez et al. 2010).

The genus *Cephenemyia* contains eight species: *C. auribarbis* (Meigen, 1824), *C. ulrichii* (Brauer, 1863), *C. stimulator* (Hunter, 1916), *C. trompe* (Modeer, 1786), *C. jellisoni* (Townsend, 1941), *C. pratti* (Hunter, 1916), *C. apicata* (Bennett and Sabrosky, 1962), and *C. phorbifer* (Clark, 1815). These species parasitize various cervid hosts (family Cervidae): *C. auribarbis* and *C. stimulator* parasitize red deer *Cervus elaphus* (Linnaeus, 1758) and roe deer *Capreolus capreolus* (Linnaeus, 1758), respectively; *C. ulrichii* is found in moose *Alces alces* (Linnaeus, 1758); *C. trompe* parasitizes both moose and reindeer *Rangifer tarandus* (Linnaeus, 1758); *C. jellisoni* has been reported in white-tailed deer *Odocoileus virginianus* (Zimmermann, 1780), moose, elk *Cervus canadensis* (Erxleben, 1777), and mule deer *Odocoileus hemionus* (Rafinesque, 1817); *C. pratti* has been found in white-tailed deer and mule deer; *C. apicata* in mule deer; and *C. phorbifer* in white-tailed deer (Morrondo et al. 2021).

The genus *Pharyngomyia* includes two species: *P. dzerenae* (Grunin, 1950) and *P. picta* (Meigen, 1824). The former parasitizes Mongolian gazelle *Procapra gutturosa* (Pallas, 1777) (Pape 2006), while the latter, which is widely distributed throughout Europe and central Asia, parasitize red deer, sika deer *Cervus nippon* (Temminck, 1838), roe deer, fallow deer *Dama dama* (Linnaeus, 1758), and moose (Colwell 2001; Sreejith et al. 2012). Concomitant infestations by both *C. auribarbis* and *P. picta* in red deer and fallow deer have been reported (Vicente et al. 2004; Leitner et al. 2016).

The genome of Oestridae species remains poorly studied. Most of the available data are related to the characterization and analysis of the molecular markers used to identify the larvae of these species, which are generally morphologically very similar (Moreno et al. 2015; de la Fuente et al. 2021). The specific identification of Oestridae larvae is challenging, largely due to interspecific morphological similarities, sample degradation, and the scarcity of diagnostic morphobiometric traits. Thus, the mitogenome marker (cytochrome c oxidase subunit I gene (*cox1*), nuclear markers (28S gene and internal transcribed spacers (ITS) of rDNA), and the complete mitogenome have been characterized and are now used in population genetics and phylogenetic analyses (de la Fuente et al. 2021).

The analysis of complete mitogenomes has proven to be a highly valuable tool in the phylogenetic reconstruction of various groups of organisms, given that the mitogenome is only inherited maternally. To date, the complete mitogenome has been reported for only 18 oestrid species. The characterization of additional mitogenomes, along with comparative analyses, may provide new molecular markers that will help determine the phylogenetic relationships between Oestridae genera and species (Li et al. 2020). For species of the genera *Pharyngomyia* and *Cephenemyia*, molecular markers such as *cox1* (from the mitogenome) and sequences of the 28S, 18S, ITS1, and ITS2 of the nuclear ribosomal RNA genes (rDNAs) are already available (de la Fuente et al. 2021). In addition, the mitogenomes of *C. stimulator* (Aleix-Mata et al. 2021a) and *C*. *trompe* (Li et al. 2020) are also available for the genus *Cephenemyia*.

The main objective of the present study was to describe the complete mitogenomes of three Oestrinae species, *Cephenemyia auribarbis*, *C. ulrichii*, and *Pharyngomyia picta*. In addition, we compared these mitogenomes with those from other Oestrinae species available in GenBank.

## Material and method

### Larvae collection and storage

Larvae of *Cephenemyia auribarbis* collected from red deer (*Cervus elaphus*) from Barcelona (Spain), of *Cephenemyia ulrichii* from moose (*Alces alces*) from Kirov (Russia), and of *Pharyngomyia picta* from red deer from the Sierras de Cazorla, Segura and Las Villas Natural Park (Spain) were used for this study. Larvae were fixed individually in 70–90% ethanol and stored at 4 °C and then identified on the basis of the shape of their posterior peritremes and dorsal and ventral spinulation (Zumpt 1965; Colwell 2006). Vouchers were deposited in the Department of Experimental Biology at the University of Jaén (Spain).

### Extraction of genomic DNA and illumina sequencing

Genomic DNAs were extracted using the ZR Tissue & Insect DNA MiniPrep kit (Zymo Research). The total genomic DNA of one larva of *C. auribarbis*, two larvae of *C. ulrichii*, and two larvae of *P. picta* were sequenced using the Illumina^®^ Hiseq™ 2000 or Illumina NovaSeq X Plus platform by Novogene (UK). The genomic DNA of one *C. ulrichii* larva and two *P. picta* larvae was used to construct a library of 350-bp-length fragments; a total of 7 Gb of paired-end reads (2 × 150 bp) were obtained from *P. picta* samples and of 8 Gb from *C. ulrichii* samples. The genomic DNA of one *C. ulrichii* larva and one *C. auribarbis* larva were used to construct a library of 750-bp-length fragments; in all, 2 Gb of paired-end reads (2 × 250 bp) were obtained.

### Mitogenome assembly and annotation

Raw reads were quality-trimmed, and sequencing adapters were removed using Trimmomatic v.0.36 (Bolger et al. 2014). A set of around 1.5 million clean reads for each species was employed for mitogenome assembly via NOVOplasty v.3.7 (Dierckxsens et al. 2017) using the mitogenomes of *C. stimulator* (Aleix-Mata et al. 2021a) as a reference. Several k-mers were tested for each mitogenome assembly and the best one in terms of mitogenome completeness was chosen for downstream analyses. The resulting mitogenomes were annotated with MITOS (http://mitos.bioinf.uni-leipzig.de) (Bernt et al. 2013) and tRNAscan-SE (http://lowelab.ucsc.edu/tRNAscan-SE) (Lowe and Eddy 1997). The annotations of protein coding genes (PCGs) and the transfer RNAs (tRNAs) and ribosomal RNA genes (rRNAs) were manually refined by comparison with the available mitogenomes of Oestridae species. The circular representation of the assembled mitogenomes was created using the OrganellarGenomeDRAW tool (https://chlorobox.mpimp-golm.mpg.de/OGDraw.html) (Greiner et al. 2019). Pairwise identities were calculated using Clustal Omega (https://www.ebi.ac.uk/Tools/msa/clustalo/) (Sievers and Higgins 2021; Madeira et al. 2022). The nucleotide bias was calculated from the base composition of the mitogenomes using the following formulas: AT-skew: A-T/A + T and GC-skew: G-C/G + C.

### Phylogenetic analysis

For the phylogenetic analysis, we employed the complete mitogenomes of 18 Oestridae species available from GenBank and the mitogenomes described in this study, with the mitogenome of *Sarcophaga tuberosa* (Pandellé, 1896) (family Sarcophagidae) as an outgroup (Supplementary Table S1).

Sequences of the complete mitogenomes were aligned using Clustal omega (https://www.ebi.ac.uk/Tools/msa/clustalo/) (Sievers and Higgins 2021; Madeira et al. 2022), and poorly aligned positions and divergent regions were removed using the Gblocks program v 0.91.1 (https://ngphylogeny.fr/tools/tool/276/form) (Talavera and Castresana 2007). Several sequence fragments were removed with the Gblocks program, specifically, approximately 190 bp from the end of the 12 S rRNA gene, the entire D-loop region, and several small fragments of variable length at various locations in the mitogenome. The length of the aligned sequences after running Gblocks was 14,096 bp. The nucleotide substitution models were evaluated using MEGA version 11 (Tamura et al. 2021). Phylogenetic relationships were reconstructed using the Maximum Likelihood (ML) method (Nei and Kumar 2000) implemented in MEGA version 11 (Tamura et al. 2021). Node supports were assessed with 1000 bootstrap replicates.

In addition, a further phylogenetic analysis was performed with different partitions and substitution models for the genes following the same approach as in Lorite et al. (2025). Briefly, all the PCGs were aligned using MAFFT (Katoh and Standley 2013) v7.453, and the phylogenetic relationships were inferred via the Maximum Likelihood partitioned method (Chernomor et al. 2016) in IQTree2 (Minh et al. 2020) using different models for each gene (Supplementary Table S2). The best model for each gene was calculated with ModelFinder (Kalyaanamoorthy et al. 2017), with 1000 ultrafast bootstrap replicates (Hoang et al. 2018) to assess branch support.

## Results and discussion

In addition to the description of the mitogenomes of *C. auribarbis*, *C. ulrichii*, and *P. picta*, we performed a comparative analysis with the mitogenomes of 18 species of Oestridae available in GenBank (see Phylogenetic analysis in the Material and Methods section).

### Genome organization and nucleotide composition

Five mitogenomes were assembled and annotated, one from each of two larvae of *P. picta* and two larvae of *C. ulrichii* and one from a larva of *C. auribarbis* (Genbank accession numbers: PV856279-PV856283) (Tables 1, 2 and 3). The two mitogenomes of *P. picta* had the same length but differed in their sequences, while the two mitogenomes of *C. ulrichii* differed in length and sequence due in both cases to base substitutions and insertions/deletions (Tables 1 and 2). The length of the described mitogenomes lie in the range of the previously described Oestridae mitogenomes, which vary in length between 14,854 bp in *G. inermis* and *G. haemorrhoidalis* (Yan et al. 2019) and 16,769 bp in *Oestrus* sp. (Aleix-Mata et al. 2025) (Supplementary Table S3).

**Table 1:** Gene organization of the two *Pharyngomyia picta *mitogenomes

Gene	Start position	Stop position	Length (bp)	Intergenic nucleotides (bp)	Anticodon	Start codon	Stop codon	Strand
*trnI*	1	64	64	−3	GAT			H
*trnQ*	62	130	69	−1	TTG			L
*trnM*	130	197	68	0	CAT			H
*nad2*	198	1,214	1,017	−2		ATC	TAA	H
*trnW*	1,213	1,279	67	25	TGA			H
*trnC*	1,305	1,368	64	47–55	GCA			L
*trnY*	1,416-1,424	1,480-1,488	65	−2	GTA			L
*cox*1	1,479-1,487	3,012 − 3,020	1,534	0		TCG	T--	H
*trnL*(UUR)	3,013 − 3,021	3,078 − 3,086	66	6	TAA			H
*cox*2	3,085 − 3,093	3,771-3,782	687–690	5		ATG	TAA	H
*trnK*	3,777-3,788	3,847-3,858	71	−1	CTT			H
*trnD*	3,847-3,858	3,912-3,923	66	0	GTC			H
*atp8*	3,913-3,924	4,077 − 4,088	165	−7		ATC/ATT	TAA	H
*atp6*	4,071 − 4,082	4,748-4,759	678	−1		ATG	TAA	H
*cox*3	4,748-4,759	5,536-5,547	789	6		ATG	TAA	H
*trnG*	5,543-5,554	5,611-5,622	69	0	TCC			H
*nad3*	5,612-5,623	5,965-5,976	354	1		ATC	TAA	H
*trnA*	5,967-5,978	6,031 − 6,042	65	−1	TGC			H
*trnR*	6,031 − 6,042	6,093 − 6,104	63	1	TCG			H
*trnN*	6,095 − 6,106	6,158-6,169	64	0	GTT			H
*trnS*(AGN)	6,159-6,170	6,226-6,237	68	17	GCT			H
*trnE*	6,244-6,255	6,308-6,319	65	18	TTC			H
*trnF*	6,327-6,338	6,393-6,404	67	1	GAA			L
*nad*5	6,395-6,406	8,126-8,137	1,732	−1		ATT	T--	L
*trnH*	8,126-8,137	8,193-8,204	68	0	GTG			L
*nad4*	8,194-8,205	9,532-9,543	1,339	−7		ATG	T--	L
*nad4L*	9,526-9,537	9,822-9,833	297	20		ATG	TAA	L
*trnT*	9,843-9,860	9,907-9,924	65	0	TGT			H
*trnP*	9,908-9,925	9,973-9,990	66	2	TGG			L
*nad*6	9,976-9,993	10,500 − 10,517	525	−1		ATT	TAA	H
*cob*	10,500 − 10,517	11,636 − 11,653	1,137	−2		ATG	TAG	H
*trnS*(UCN)	11,635 − 11,652	11,702 − 11,719	68	16	TGA			H
*nad1*	11,719 − 11,736	12,666 − 12,683	948	1		TTG	TAG	L
*trnL*(CUN)	12,668 − 12,685	12,731 − 12,748	64	0	TAG			L
*rrnL*	12,732 − 12,749	14,062 − 14,077	1,331-1,329	0				L
*trnV*	14,063 − 14,078	14,134 − 14,149	72	0	TAC			L
*rrnS*	14,135 − 14,150	14,929 − 14,944	795	0				L
D-loop	14,930 − 14,945	16,651	1,722-1,707					H

**Table 2:** Gene organization of the two *Cephenemyia ulrichii* mitogenomes

Gene	Start position	Stop position	Length (bp)	Intergenic nucleotides (bp)	Anticodon	Start codon	Stop codon	Strand
*trnI*	1	65	65	−3	GAT			H
*trnQ*	63	131	69	−1	TTG			L
*trnM*	131	198	68		CAT			H
*nad2*	199	1,215	1,017	−2		ATT	TAA	H
*trnW*	1,214	1,281	68	37–55	TGA			H
*trnC*	1,319-1,337	1,381-1,399	63	21 − 20	GCA			L
*trnY*	1,403-1,420	1,467-1,484	65	−2	GTA			L
*cox*1	1,466-1,483	2,999-3,016	1,534			TCG	T--	H
*trnL*(UUR)	3,000–3,017	3,065 − 3,082	66	27	TAA			H
*cox*2	3,093 − 3,110	3,776-3,793	684	26		ATG	TAA	H
*trnK*	3,803-3,820	3,873-3,890	71		CTT			H
*trnD*	3,874-3,891	3,936-3,953	63	1	GTC			H
*atp8*	3,938-3,955	4,099 − 4,116	162	−7		ATT	TAA	H
*atp6*	4,093 − 4,110	4,770-4,787	678	−1		ATG	TAA	H
*cox*3	4,770-4,787	5,558-5,575	789	6		ATG	TAA	H
*trnG*	5,565-5,582	5,629-5,646	65		TCC			H
*nad3*	5,630-5,647	5,983-6,000	354	−2		ATT	TAG	H
*trnA*	5,982-5,999	6,046 − 6,063	65	−1	TGC			H
*trnR*	6,046 − 6,063	6,107-6,124	62		TCG			H
*trnN*	6,108-6,125	6,171-6,188	64		GTT			H
*trnS*(AGN)	6,172-6,189	6,239-6,256	68		GCT			H
*trnE*	6,240-6,257	6,305-6,322	66	18	TTC			H
*trnF*	6,324-6,341	6,391-6,408	68		GAA			L
*nad*5	6,392-6,409	8,123-8,140	1,732			ATT	T--	L
*trnH*	8,124-8,141	8,188-8,205	65		GTG			L
*nad4*	8,189-8,206	9,527-9,544	1,339	−7		ATG	T--	L
*nad4L*	9,521-9,538	9,814-9,831	294	7		ATG	TAA	L
*trnT*	9,822-9,839	9,884-9,901	63		TGT			H
*trnP*	9,885-9,902	9,950-9,967	66	2	TGG			L
*nad*6	9,953-9,970	10,477 − 10,494	525	−1		ATT	TAA	H
*cob*	10,477 − 10,494	11,613 − 11,630	1,137	17		ATG	TAA	H
*trnS*(UCN)	11,631 − 11,648	11,698 − 11,715	68	16	TGA			H
*nad1*	11,715 − 11,732	12,662 − 12,679	948	2		ATG	TAG	L
*trnL*(CUN)	12,665 − 12,682	12,729 − 12,746	65	1	TAG			L
*rrnL*	12,731 − 12,748	14,059 − 14,078	1,329-1,331					L
*trnV*	14,060 − 14,079	14,131 − 14,150	72		TAC			L
*rrnS*	14,132 − 14,151	14,919 − 14,938	788					L
D-loop	14,920 − 14,939	16,446 − 16,466	1,527-1,528					H

**Table 3:** Gene organization of the *Cephenemyia auribarbis* mitogenome

Gene	Start position	Stop position		Length (bp)		Intergenic nucleotides (bp)			Anticodon	Start codon		Stop codon		Strand			
*trnI*			1		66		66	−3			GAT						H
*trnQ*			64		132		69	−1			TTG						L
*trnM*			132		199		68	0			CAT						H
*nad2*			200		1,216		1,017	−2					ATT		TAA		H
*trnW*			1,215		1,282		68	59			TGA						H
*trnC*			1,342		1,404		63	26			GCA						L
*trnY*			1,431		1,494		64	−2			GTA						L
*cox*1			1,493		3,026		1,534	0					TCG		T--		H
*trnL*(UUR)			3,027		3,092		66	4			TAA						H
*cox*2			3,097		3,780		684	8					ATG		TAA		H
*trnK*			3,789		3,858		70	0			CTT						H
*trnD*			3,859		3,923		65	0			GTC						H
*atp8*			3,924		4,085		162	−7					ATT		TAA		H
*atp6*			4,079		4,756		678	−1					ATG		TAA		H
*cox*3			4,756		5,544		789	4					ATG		TAA		H
*trnG*			5,549		5,614		66	0			TCC						H
*nad3*			5,615		5,968		354	−2					ATT		TAG		H
*trnA*			5,967		6,031		65	−1			TGC						H
*trnR*			6,031		6,092		62	0			TCG						H
*trnN*			6,093		6,156		64	0			GTT						H
*trnS*(AGN)			6,157		6,224		68	0			GCT						H
*trnE*			6,225		6,289		65	18			TTC						H
*trnF*			6,308		6,372		65	0			GAA						L
*nad*5			6,373		8,104		1,732	0					ATT		T--		L
*trnH*			8,105		8,168		64	0			GTG						L
*nad4*			8,169		9,507		1,339	−7					ATG		T--		L
*nad4L*			9,501		9,794		294	9					ATG		TAA		L
*trnT*			9,804		9,866		63	0			TGT						H
*trnP*			9,867		9,932		66	2			TGG						L
*nad*6			9,935		10,459		525	−1					ATT		TAA		H
*cob*			10,459		11,595		1,137	6					ATG		TAA		H
*trnS*(UCN)			11,602		11,668		67	16			TGA						H
*nad1*			11,685		12,629		945	2					ATG		TAG		L
*trnL*(CUN)			12,632		12,696		65	0			TAG						L
*rrnL*			12,697		14,019		1,323	0									L
*trnV*			14,020		14,091		72	0			TAC						L
*rrnS*			14,092		14,879		788	0									L
D-loop			14,880		16,372		1,493										H

**Fig. 1:**
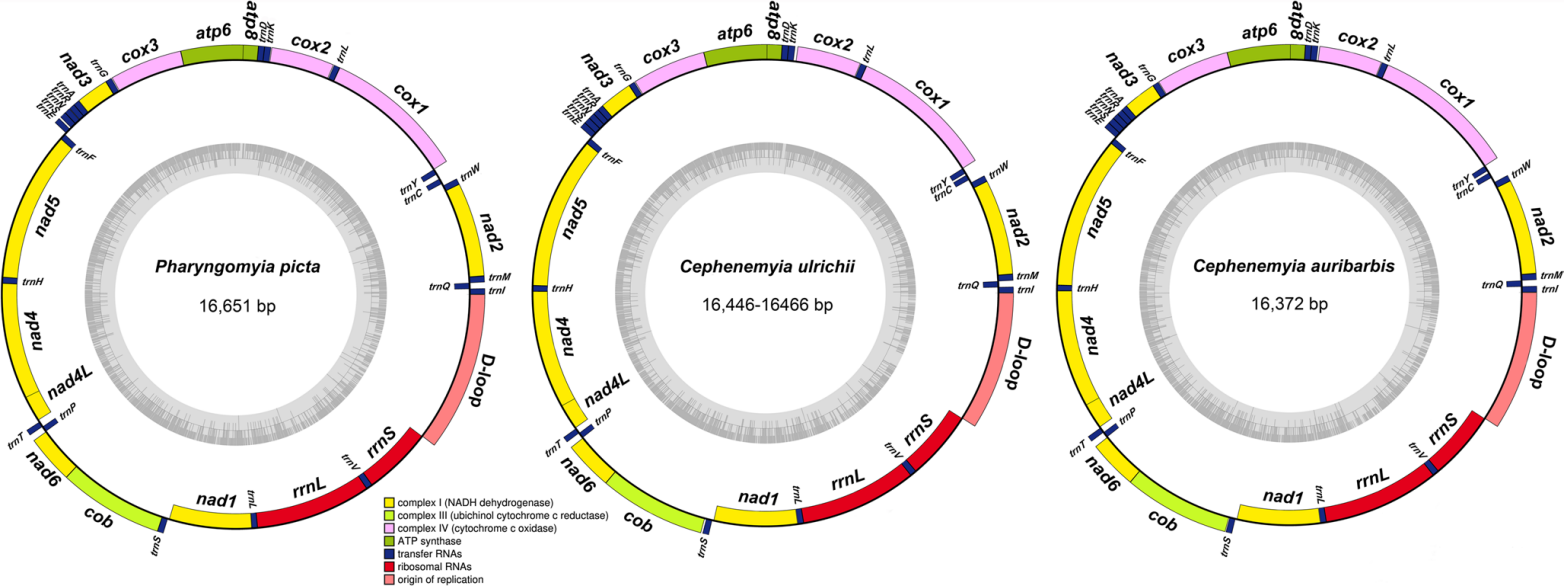
Map of the mitogenomes of *Pharyngomyia picta*, *Cephenemyia ulrichii*, and *Cephenemyia auribarbis*. Genes encoded by the heavy strand (H) are shown outside the circle and those encoded by the light strand (L) are shown inside the circle. The inner ring shows the GC content of this genome

The gene organization found in our study is identical to that of the mitogenomes of all previously described Oestridae species. It contains the typical 13 protein-coding genes (PCGs), two rRNA genes, 22 tRNA genes, and a control region (CR or D-loop region) (Fig. 1; Tables 1, 2 and 3) (Gao et al. 2016; Li et al. 2020; Aleix-Mata et al. 2021a, b, 2025). Most of the genes are encoded on the heavy strand, and just four PCGs (*nad1*, *nad4*, *nad4L*, and *nad*5), the two rRNA genes, and eight of the tRNAs are encoded on the light strand (Fig. 1; Tables 1, 2 and 3) (Supplementary Table S3) (Gao et al. 2016; Li et al. 2020; Aleix-Mata et al. 2021a, b, 2025).

The base composition and the A + T content of the three complete mitogenomes described here were very similar to the average base composition and A + T content of all previously analyzed Oestridae mitogenomes (Supplementary Table S4). A high A + T bias content is a general characteristic of the mitogenomes of the Oestridae and other dipteran species (Junqueira et al. 2004; Aleix-Mata et al. 2025). In addition, Oestridae mitogenomes show a slight bias toward A in relation to T (positive AT-skew average 0.06) and are clearly biased toward C in relation to G (negative GC-skew average − 0.28). These biases have been described in the mitogenomes of other dipteran species (Junqueira et al. 2004; Aleix-Mata et al. 2025).

### Protein-Coding genes (PCGs) and codon usage

The 13 PCGs encoded by the five described mitogenomes range in size from 294 bp (*nad4L*) to 1534 bp (*cox1*). The size of most of the genes was identical in the five mitogenomes, the exceptions being *cox2*, *atp8*, *nad1*, and *nad4L*. The two mitogenomes of *P. picta* differed in the size of *cox2* (687 bp and 690 bp) caused by a three-bp deletion at the 3´ end that does not change the reading frame.

In general, when comparing the PCGs of all Oestridae mitogenomes, only three genes (*cox1*, *cox3*, and *cob*) share the same length in all species. The remaining genes vary in length between the species, although this variation does not affect all the genes to the same extent (Supplementary Table S3).

The comparison of the 13 PCG genes from all mitogenomes described here and those from the 18 *Oestridae* species demonstrates that, in terms of the nucleotide sequences, the most conserved genes were *cox1*, *cox3*, and *nad1*, and the least conserved were *atp8* and *nad6*.

Regarding the start codons, the two *P. picta* mitogenomes shared the same start codons in all genes except *atp8*, which initiated with either ATC or ATT (Table 1). However, the three *Cephenemyia* mitogenomes described here shared identical start codons in all genes, except the *nad3*, which used ATT in *C. ulrichii* and ATC in *C. auribarbis* (Tables 2 and 3). It is interesting to note that in all the described mitogenomes, the *cox1* gene uses the TCG start codon, which differs from the standard invertebrate mitogenome code but is a common feature in this gene in the mitogenomes of the species of the family Oestridae. This feature has also been described in the mitogenomes of species from the families Calliphoridae, Tephritidae, and Culicidae (Gao et al. 2016; Junqueira et al. 2004).

In the 23 mitogenomes analyzed from the Oestridae family, seven genes conserved the same start codons: genes *cox2*, *atp6*, *cox3*, *nad4*, *nad4L*, while *cob* used ATG and *cox1* the codon TCG. In the remaining genes, the start codon varied, although in general one start codon was found to be the most frequent (Supplementary Table S3). In general, in Oestridae species analyzed, of the start codons (ATG, ATA, ATT, ATC, TTG, GTG, and TCG) the most commonly used are ATG (49.50%) and ATT (29.76%) and the least used are GTG (0.67%) and TTG (1.34%).

In relation to the stop codons, 10 genes in the *C. ulrichii*, *C. auribarbis* (*C. trompe* and *C. stimulator*) and *P. picta* mitogenomes used the same stop codons, the exceptions being the gene *nad3*, which used TAG in *C. ulrichii*, *C. auribarbis*, and *C. stimulator*, T– in *C. trompe*, and TAA in *P. picta*, and the gene *cob*, which used TAA in all the mitogenomes of *Cephenemyia* species and TAG in *P. picta* mitogenome.

In the 23 Oestridae mitogenomes analyzed, only four genes shared the same stop codon, while the remaining genes varied in their stop codons (Supplementary Table S3). Overall, the frequencies of stop-codon usage were TAA (58.19%), the incompletes T– (22.74%), TA- (2.01%), and TAG (17.06%), whereas the AGA codon was never used.

### Ribosomal RNA genes and transfer RNA

In the mitogenomes of the Oestridae species, *rrnL* and *rrnS* are located between the D-loop and *trnL*, and are separated by *trnV*. The *rrnS* length was 795 bp in *P. picta* and 788 bp in *C. ulrichii* and *C. auribarbis* (Tables 1, 2 and 3) but was 789 bp in *C. trompe* and *C. stimulator* (Li et al. 2020; Aleix-Mata et al. 2021a). Variation in this gene in Oestridae species ranges between 597 bp in *Gasterophilus nigricornis* (Yan et al. 2019) and 795 bp in *P. picta* (Supplementary Table S3).

The *rrnS* in the mitogenomes of *C. ulrichii*, *C. auribarbis*, and *P. picta* have an A + T content of 78.40, 79.10, and 78%, respectively. In general, the 23 Oestidae mitogenomes analyzed were A + T rich (with an A + T content average of 76.08%) (Zhang et al. 2016; Li et al. 2020).

The length of the *rrnL* gene was 1,323 bp in *C. auribarbis*, and 1,329 bp and 1,331 bp in the mitogenomes of *C. ulrichii* and *P. picta*, respectively (Tables 1 and 2, and 3). The variation in this gene in Oestridae species ranged between 1296 bp in an undescribed species of *Hypoderma* sp. (Zhang et al. 2025) and 1331 bp in *P. picta* and *C. ulrichii* (Supplementary Table S3).

The *rrnL* in the *C. auribarbis* mitogenome had an A + T content of 82.10%. The two *C. ulrichii* mitogenomes had 81.40% and 81.30%, while the two *P. picta* mitogenomes had 81.40% and 81.50%. In general, the 23 analyzed Oestridae mitogenomes were A + T rich (with an A + T content average of 78.99%) like other dipteran species (Weigl et al. 2010; Zhang et al. 2014; Li et al. 2020).

All 22 typical tRNAs were identified in the mitogenomes described here and in all the other mitogenomes of Oestridae species. They had highly conserved lengths ranging from 61 bp (*trnC*) to 72 bp (*trnV*) (Supplementary Table S3), which add up on average to 1457 bp in length.

### Non-Coding regions

The mitogenome genes of the species described here overlap or have intergenic non-coding nucleotides in several positions. The overlapping nucleotides vary from one to seven in 10 to 12 positions depending on the mitogenome; the intergenic nucleotides vary from 1 to 59 bp in 11 to 14 positions between genes (Tables 1, 2 and 3).

The D-loop region, located between the *rrnS* gene and *trnI* gene, varied in length: 1527 bp and 1528 bp in *C. ulrichii*, 1493 bp in *C. auribarbis*, and 1707 bp and 1722 bp in *P. picta* (Tables 1 and 2, and 3). In Oestridae mitogenomes, the D-loop region length varies between 58 bp in *Gasterophilus inermis* and *G. haemorrhoidalis* (Yan et al. 2019) and 1,722 bp in *P. picta* (Supplementary Table S3).

The D-loop region is the most variable region of the Oestridae mitogenomes and the variation in length and in sequences makes sequence alignment very difficult. Indeed, the average of identity is 51.48%. The D-loop region in all the analyzes of Oestridae species has an average of 80.58% of A + T content, ranging from 62.1% in *G. inermis* and *G. haemorrhoidalis* (Yan et al. 2019) to 89.5% in *Hypoderma bovis* (Chen et al. 2023).

### Phylogenetic analysis

**Fig. 2:**
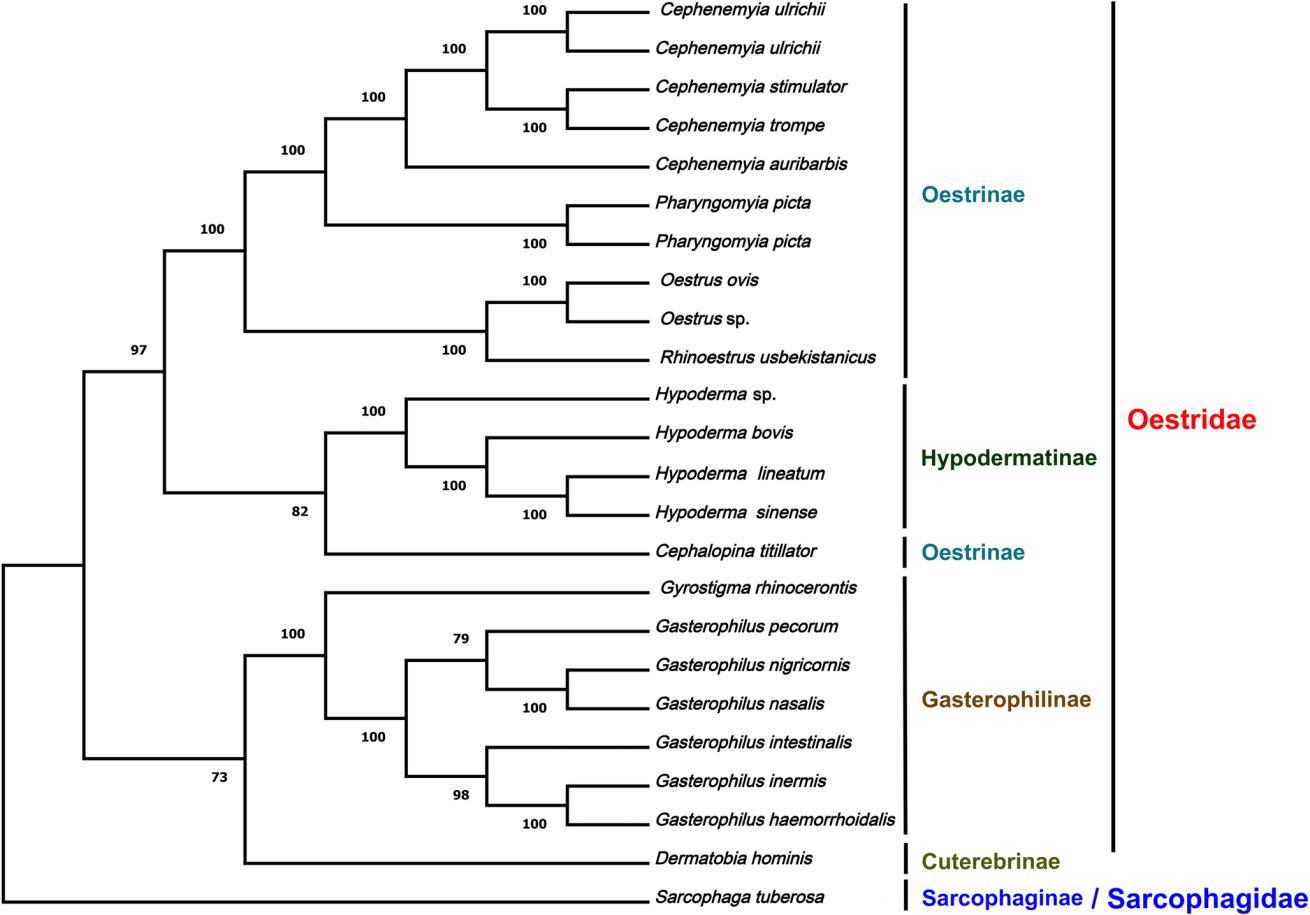
Phylogenetic tree inferred with the Maximum Likelihood partitioned method in IQTree2 using different models for each PCG gene sequences of the mitogenomes of *Pharyngomyia picta*, *Cephenemyia ulrichii*,* Cephenemyia auribarbis*, and 18 other Oestridae species. The data at the nodes correspond to Bootstrap supports of 1000 replicates and represent the statistical support

The phylogenetic relationships were studied with a Maximum-Likelihood (ML) tree using the complete mitogenomes (Supplementary Fig. S1) and with different partitions and substitution models for the PCGs (Fig. 2). In total 24 mitogenomes were included in the analyses, five of which were those of *C. ulrichii*, *C. auribarbis*, and *P. picta* newly described here. The remaining 18 mitogenomes from Oestridae species were available in GenBank; the mitogenome of *Sarcophaga tuberosa* (Diptera: Sarcophagidae) was included as an outgroup (Kai et al. 2019) (Supplementary Table S1).

The same topology and almost identical support were obtained either in the complete mitogenomes tree (Supplementary Fig. S1) or the PCGs partitioned tree (Fig. 2), the only difference being the association of *C. titillator* with the Gasterophilinae in the former and with Hypodermatinae in the latter. The four subfamilies of Oestridae, including their respective genera and species, are grouped into clear well-differentiated branches with high statistical support. The species of the genus *Cephenemyia* grouped into one cluster, with *Pharyngomyia* as a sister genus, and clustered with another group including the genera *Oestrus* and *Rhinoestrus*. This association between the genera *Oestrus*, *Rhinoestrus*, and *Cephenemyia* has been previously documented in other phylogenetic analyses (Pape 2001; Moreno et al. 2015; Pape et al. 2017; Aleix-Mata et al. 2025). In general, our data agree with previous analyses of the phylogenetic relationships between genera in the family Oestridae (Moreno et al. 2015; de la Fuente et al. 2021; Aleix-Mata et al. 2025). However, *Cephalopina titillator* (Clark, 1816) from the Oestrinae family clusters with the stomach bot flies (Gasterophilinae) or with warble flies (Hypodermatinae), thereby splitting this subfamily Oestrinae into two separate groups. Phylogenies carried out with mitogenome genes demonstrated both the association between *C. titillator* and Gasterophilinae (Shamsi et al. 2023; Yao et al. 2025) and the association between *C. titillator* and species of Oestrinae (Li et al. 2020; Chen et al. 2023).

## Conclusions

This study presents the first complete description and characterization of the mitogenomes for *Cephenemyia auribarbis*, *C. ulrichii*, and *Pharyngomyia picta*. Our comparative analysis shows that these mitogenomes have a highly conserved structure and gene order, similar to other Oestridae species. While protein-coding genes and ribosomal RNAs are highly conserved, significant intraspecific variability was found in the length and sequence of the D-loop region, confirming it as the most divergent part of the bot fly mitogenome. Phylogenetic analysis of both complete mitogenomes and partitioned protein-coding genes clearly resolved the evolutionary relationships of these species. Our results confirm the monophyly of *Cephenemyia* and place *Pharyngomyia* as its closest sister genus, forming a well-supported clade with *Oestrus* and *Rhinoestrus*. These findings highlight the value of mitogenome data in understanding Oestridae evolution. The described mitogenomes provide essential resources for future studies on taxonomy, population genetics, and molecular evolution of these specialized parasites.

## Supplementary Information

Below is the link to the electronic supplementary material.

Supplementary Material 1

## Data Availability

The datasets generated during and analyzed during this study are available in the GenBank repository, the Sequence Read Archives (SRAs) under the BioProject accession PRJNA1322541 and the BioSample accessions: SAMN51250128, SAMN51250129, SAMN51250130, SAMN51250131 and SAMN51250132.
