# Supplementary material for: Complete mitogenomes of two species of Cephenemyia and Pharyngomyia picta, and a comparison with other Oestrinae
Source: Parasitol Res. 2026 Jan 13;125(1):9. doi: 10.1007/s00436-025-08623-9 (PMC12799752; doi:10.1007/s00436-025-08623-9)

**Supplementary Data**

| **Supplementary Table S1**. List of species of Oestroidea analysed and used for phylogenetic analysis, accession number and reference for the mitogenome description are included. | | | | | | |
| --- | --- | --- | --- | --- | --- | --- |
| Superfamily | Family | Subfamily | Genus | Species | Accession number | Reference |
| Oestroidea | Oestridae | Cuterebrinae | *Dermatobia* | *D. hominis* (Linnaeus, 1781) | AY463155 | Azeredo-Espin et al., 2004 |
|  |  | Gasterophilinae | *Gasterophilus* | *G. haemorrhoidalis* (Linnaeus, 1758) | MG920502 | Yan et al., 2019 |
|  |  |  |  | *G. inermis* (Brauer, 1859) | MG920503 | Yan et al., 2019 |
|  |  |  |  | *G. intestinalis* (De Geer, 1776) | KU236026 | Gao et al., 2016 |
|  |  |  |  | *G. nasalis* (Linnaeus, 1758) | MG920505 | Yan et al., 2019 |
|  |  |  |  | *G. nigricornis* (Loew, 1863) | MG920506 | Yan et al., 2019 |
|  |  |  |  | *G. pecorum* (Fabricius, 1794) | KU578262 | Zhang et al., 2016 |
|  |  |  | *Gyrostigma* | *G. rhinocerontis* (Owen, 1830) | MK045312 | Yan et al., 2019 |
|  |  | Hypodermatinae | *Hypoderma* | *H. bovis* Linnaeus, 1758 | NC_080982 | Chen et al., 2023 |
|  |  |  |  | *H. lineatum* (De Villiers, 1789) | GU584123 | Weigl et al., 2010 |
|  |  |  |  | *H. sp* | PP078819 | Zhang et al., 2025 |
|  |  |  |  | *H. sinense* Pleske, 1926 | NC_071819 | Tang et al., 2022 direct submission to GenBank |
|  |  | Oestrinae | *Cephalopina* | *C. titillator* (Clark, 1816) | MN833258 | Li et al., 2020 |
|  |  |  | *Cephenemyia* | *C. auribarbis* (Meigen, 1824) | PV856281 | This work |
|  |  |  |  | *C. stimulator* (Hunter, 1916) | NC_059850 | Aleix-Mata et al., 2021a |
|  |  |  |  | *C. trompe* (Modeer, 1786) | MN814272 | Li et al., 2020 |
|  |  |  |  | *C. ulrichii* (Brauer, 1863) | PV856279 | This work |
|  |  |  |  | *C. ulrichii* (Brauer, 1863) | PV856280 | This work |
|  |  |  | *Oestrus* | *O. ovis* Linnaeus, 1758 | NC_059851 | Aleix-Mata et al., 2021b |
|  |  |  |  | *O.* sp. | PQ516713 | Aleix-Mata et al., 2025 |
|  |  |  | *Pharyngomyia* | *P. picta* (Meigen, 1824) | PV856282 | This work |
|  |  |  |  | *P. picta* (Meigen, 1824) | PV856283 | This work |
|  |  |  | *Rhinoestrus* | *R. usbekistanicus* Gan, 1947 | MN833259 | Li et al., 2020 |
|  | Sarcophagidae | Sarcophaginae | *Sarcophaga* | *S. tuberosa* (Pandellé, 1896) | MK820723 | Kai et al., 2019 |

References:

Aleix-Mata G, López-Beceiro AM, Fidalgo LE, Peréz JM, Sanchéz A (2021a) The complete mitochondrial genome of *Cephenemyia stimulator* (Diptera: Oestridae). Mitochondrial DNA B Resour 6:2941–2942. <https://doi.org/10.1080/23802359.2021.1945969>

Aleix-Mata G, Peréz JM, Sánchez A. (2021b) The complete mitochondrial genome of *Oestrus ovis* (Linnaeus, 1758) (Diptera: Oestridae). Mitochondrial DNA B Resour 6:1847–1848. <https://doi.org/10.1080/23802359.2021.1934174>

Aleix-Mata G, Granados JE, Montiel EE, Caparrós N, Mora P, Rico-Porras JM, Mendoza EF, Pérez JM, Sánchez A (2025) Characterizing the complete mitochondrial genome and rDNA of *Oestrus* sp. (Diptera: Oestridae): the bot fly parasitizing the Iberian ibex, *Capra pyrenaica*. J Med Entomol 62:808-815. <https://doi.org/10.1093/jme/tjaf045>

Azeredo-Espin AM, Junqueira AC, Lessinger AC, Torres TT, Lyra ML, da Silva FR, Maia AAM (2004) The complete mitochondrial genome of the human bot fly *Dermatobia hominis* (Diptera: Oestridae). [The 2004 ESA (Entomological Society of America) Annual Meeting and Exhibition](https://esa.confex.com/esa/2004/techprogram/meeting_2004.htm). <https://esa.confex.com/esa/2004/techprogram/session_2211.htm>

Chen W, Zhang H, Meng R, Zhang X, Duo H, Guo Z, Shen X, Chen C, Li Z, Fu Y (2023) Genome-wide phylogenetic and genetic evolutionary analyses of mitochondria in *Hypoderma bovis* and *H. sinense* on the Qinghai-Tibetan Plateau. Parasitol Res 123:43. <https://doi.org/10.1007/s00436-023-08060-6>

Gao DZ, Liu GH, Song HQ, Wang GL, Wang CR, Zhu XQ (2016) The complete mitochondrial genome of *Gasterophilus intestinalis*, the first representative of the family Gasterophilidae. Parasitol Res 115*:*2573–2579. <https://doi.org/10.1007/s00436-016-5002-9>

Kai X, Shiwen W, Shang Y, Ren L, Guo Y (2019). The complete mitochondrial genome of *Sarcophaga tuberosa* (Diptera: Sarcophagidae). Mitochondrial DNA Part B 4:2757–2758. https://doi.org/10.1080/23802359.2019.1644218

Li XY, Yan LP, Pape T, Gao YY, Zhang D (2020) Evolutionary insights into bot flies (Insecta: Diptera: Oestridae) from comparative analysis of the mitochondrial genomes. Int J Biol Macromol 149:371–380. <https://doi.org/10.1016/j.ijbiomac.2020.01.249>

Weigl S, Testini G, Parisi A, Dantas-Torres F, Traversa D, Colwell DD, Otranto D (2010) The mitochondrial genome of the common cattle grub, *Hypoderma lineatum*. Med Vet Entomol 24:329–335. <https://doi.org/10.1111/j.1365-2915.2010.00873.x>.

Yan L, Pape T, Elgar M, Gao Y, Zhang D (2019) Evolutionary history of stomach bot flies in the light of mitogenomics. Syst Entomol 44:797–809. <https://doi.org/10.1111/syen.12356>

Zhang D, Yan L, Zhang M, Chu H, Cao J, Li K, Hu D, Pape T (2016) Phylogenetic inference of calyptrates, with the first mitogenomes for Gasterophilinae (Diptera: Oestridae) and Paramacronychiinae (Diptera: Sarcophagidae). Int J Biol Sci 12:489–504. <https://doi.org/10.7150/ijbs.12148>.

Zhang F, Zhang H, Li Z, Meng R, Ye P, Fu Y (2025) Evolutionary Analysis of *Hypoderma Pantholopsum* in Tibetan Antelopes on the Qinghai-Tibetan Plateau. Acta Parasitol 70:9. <https://doi.org/10.1007/s11686-024-00954-4>

| **Supplementary Table S2**. The partitioning substitution models for each PCGs dataset using 24 species for ML phylogenetic analyses. | | |
| --- | --- | --- |
| Gene | Model | |
| *atp6* | | TIM2+F+I+G4 |
| *atp8* | | HKY+F+I+G4 |
| *cob* | | TIM2+F+I+G4 |
| *cox1* | | GTR+F+I+G4 |
| *cox2* | | TIM2+F+I+G4 |
| *cox3* | | GTR+F+I+G4 |
| *nad1* | | TIM+F+I+R3 |
| *nad2* | | TIM+F+I+G4 |
| *nad3* | | TIM2+F+I+G4 |
| *nad4* | | GTR+F+R3 |
| *nad4L* | | TIM+F+R3 |
| *nad5* | | TIM+F+I+G4 |
| *nad6* | | TIM2+F+R3 |

| **Supplementary Table S3**. Gene organization of the 23 Oestridae mitogenomes | | | | | |
| --- | --- | --- | --- | --- | --- |
| Gene | Length (pb) | Length (aa) | Start  codon | Stop codon | Strand |
| *trnI* | 64-67 |  |  |  | H |
| *trnQ* | 69 |  |  |  | L |
| *trnM* | 66-69 |  |  |  | H |
| *nad2* | 1,011-1,017 | 337-338 | ATT/ATC/ATA | TAA/TAG | H |
| *trnW* | 67-68 |  |  |  | H |
| *trnC* | 61-64 |  |  |  | L |
| *trnY* | 63-67 |  |  |  | L |
| *cox*1 | 1534 | 511 | TCG | T-- | H |
| *trnL*(UUR) | 64-67 |  |  |  | H |
| *cox*2 | 681-690 | 226-229 | ATG | TAA/T-- | H |
| *trnK* | 70-71 |  |  |  | H |
| *trnD* | 63-68 |  |  |  | H |
| *atp8* | 162-165 | 53-54 | ATT/ATC/ATA | TAA | H |
| *atp6* | 648-678 | 215-225 | ATG | TAA | H |
| *cox*3 | 789 | 262 | ATG | TAA/TA- | H |
| *trnG* | 64-69 |  |  |  | H |
| *nad3* | 352-357 | 117-118 | ATT/ATC/ATA | TAA/TAG/T-- | H |
| *trnA* | 64-66 |  |  |  | H |
| *trnR* | 62-63 |  |  |  | H |
| *trnN* | 64-65 |  |  |  | H |
| *trnS*(AGN) | 68-71 |  |  |  | H |
| *trnE* | 65-67 |  |  |  | H |
| *trnF* | 63-68 |  |  |  | L |
| *nad*5 | 1,717-1,736 | 572-579 | ATT/GTG/ATG/ATA/TTG | TA-/T-- | L |
| *trnH* | 64-68 |  |  |  | L |
| *nad4* | 1,336-1,341 | 445-447 | ATG | TAA/TAG/TA-/T-- | L |
| *nad4L* | 285-301 | 94-100 | ATG | TAA/TAG/T-- | L |
| *trnT* | 62-67 |  |  |  | H |
| *trnP* | 65-68 |  |  |  | L |
| *nad*6 | 522-528 | 173-175 | ATT/ATC/ATA | TAA | H |
| *cob* | 1137 | 378 | ATG | TAA/TAG | H |
| *trnS*(UCN) | 65-69 |  |  |  | H |
| *nad1* | 939-948 | 312-315 | ATT/ATA/ATG/TTG | TAA/TAG | L |
| *trnL*(CUN) | 63-66 |  |  |  | L |
| *rrnL* | 1,296-1,331 |  |  |  | L |
| *trnV* | 72 |  |  |  | L |
| *rrnS* | 597-795 |  |  |  | L |
| D-loop | 58-1722 |  |  |  | H |
| Total | 14,854-16,769 |  |  |  |  |

| **Supplementary Table S4**. Base composition of the Oestridae mitogenomes | | | | | | | | |
| --- | --- | --- | --- | --- | --- | --- | --- | --- |
| Species | A | C | G | T | A+T | G+C | AT skew | GC skew |
| *Cephenemyia ulrichii* | 39.50 | 14.20 | 8.60 | 37.70 | 77.20 | 22.80 | 0.023 | -0.246 |
| *Cephenemyia ulrichii* | 39.40 | 14.30 | 8.60 | 37.70 | 77.10 | 22.90 | 0.022 | -0.249 |
| *Cauribarbis-consenso* | 39.50 | 14.60 | 8.60 | 37.20 | 76.70 | 23.20 | 0.030 | -0.259 |
| *Cephenemyia stimulator* | 39.50 | 14.20 | 8.30 | 37.90 | 77.40 | 22.50 | 0.021 | -0.262 |
| *Cephenemyia trompe* | 39.60 | 14.10 | 8.30 | 38.00 | 77.60 | 22.40 | 0.021 | -0.259 |
| *Oestrus ovis* | 39.60 | 15.90 | 8.30 | 36.10 | 75.70 | 24.20 | 0.046 | -0.314 |
| *Oestrus* sp. | 39.40 | 16.40 | 8.50 | 35.70 | 75.10 | 24.90 | 0.049 | -0.317 |
| *Rhinoestrus usbekistanicus* | 40.40 | 14.70 | 8.20 | 36.60 | 77.00 | 22.90 | 0.049 | -0.284 |
| *Hypoderma Lineatum* | 40.60 | 13.80 | 8.30 | 37.20 | 77.80 | 22.10 | 0.044 | -0.249 |
| *Hypoderma sinense* | 40.50 | 14.40 | 8.30 | 36.70 | 77.20 | 22.70 | 0.049 | -0.269 |
| *Hypoderma bovis* | 40.80 | 14.50 | 8.50 | 36.20 | 77.00 | 23.00 | 0.060 | -0.261 |
| *Hypoderma* sp. | 40.80 | 15.50 | 8.40 | 35.30 | 76.10 | 23.90 | 0.072 | -0.297 |
| *Cephalopina titillator* | 41.00 | 17.80 | 8.90 | 32.30 | 73.30 | 26.70 | 0.119 | -0.333 |
| *Gyrostigma rhinocerontis* | 37.40 | 19.90 | 10.20 | 32.40 | 69.80 | 30.10 | 0.072 | -0.322 |
| *Gasterophilus pecorum* | 38.40 | 18.90 | 10.30 | 32.40 | 70.80 | 29.20 | 0.085 | -0.295 |
| *Gasterophilus nigricornis* | 38.00 | 19.40 | 10.40 | 32.30 | 70.30 | 29.80 | 0.081 | -0.302 |
| *Gasterophilus nasalis* | 37.60 | 19.90 | 10.80 | 31.70 | 69.30 | 30.70 | 0.085 | -0.296 |
| *Gasterophilus intestinalis* | 38.40 | 19.50 | 10.20 | 31.90 | 70.30 | 29.70 | 0.092 | -0.313 |
| *Gasterophilus inermis* | 37.60 | 21.00 | 10.70 | 30.70 | 68.30 | 31.70 | 0.101 | -0.325 |
| *Gasterophilus haemorrhoidalis* | 37.60 | 21.10 | 10.70 | 30.70 | 68.30 | 31.80 | 0.101 | -0.327 |
| *Dermatobia hominis* | 40.60 | 13.60 | 8.60 | 37.20 | 77.80 | 22.20 | 0.044 | -0.225 |
| *Pharyngomyia picta* | 39.70 | 13.60 | 8.50 | 38.10 | 77.80 | 22.10 | 0.021 | -0.231 |
| *Pharyngomyia picta* | 39.70 | 13.60 | 8.50 | 38.20 | 77.90 | 22.10 | 0.019 | -0.231 |
| Average | 39.37 | 16.30 | 9.07 | 35.23 | 74.60 | 25.37 | 0.06 | -0.28 |

**Supplementary Figure S1**.- Maximum likelihood (ML) tree using complete mitogenomes sequences of *Pharyngomyia picta*, *Cephenemyia ulrichii, Cephenemyia auribarbis* and 18 Oestridae species. The data at the nodes correspond to Bootstrap supports of 1000 replicates and represent the statistical support for them. The tree has been performed using the nucleotide substitution model GTR+G+I.


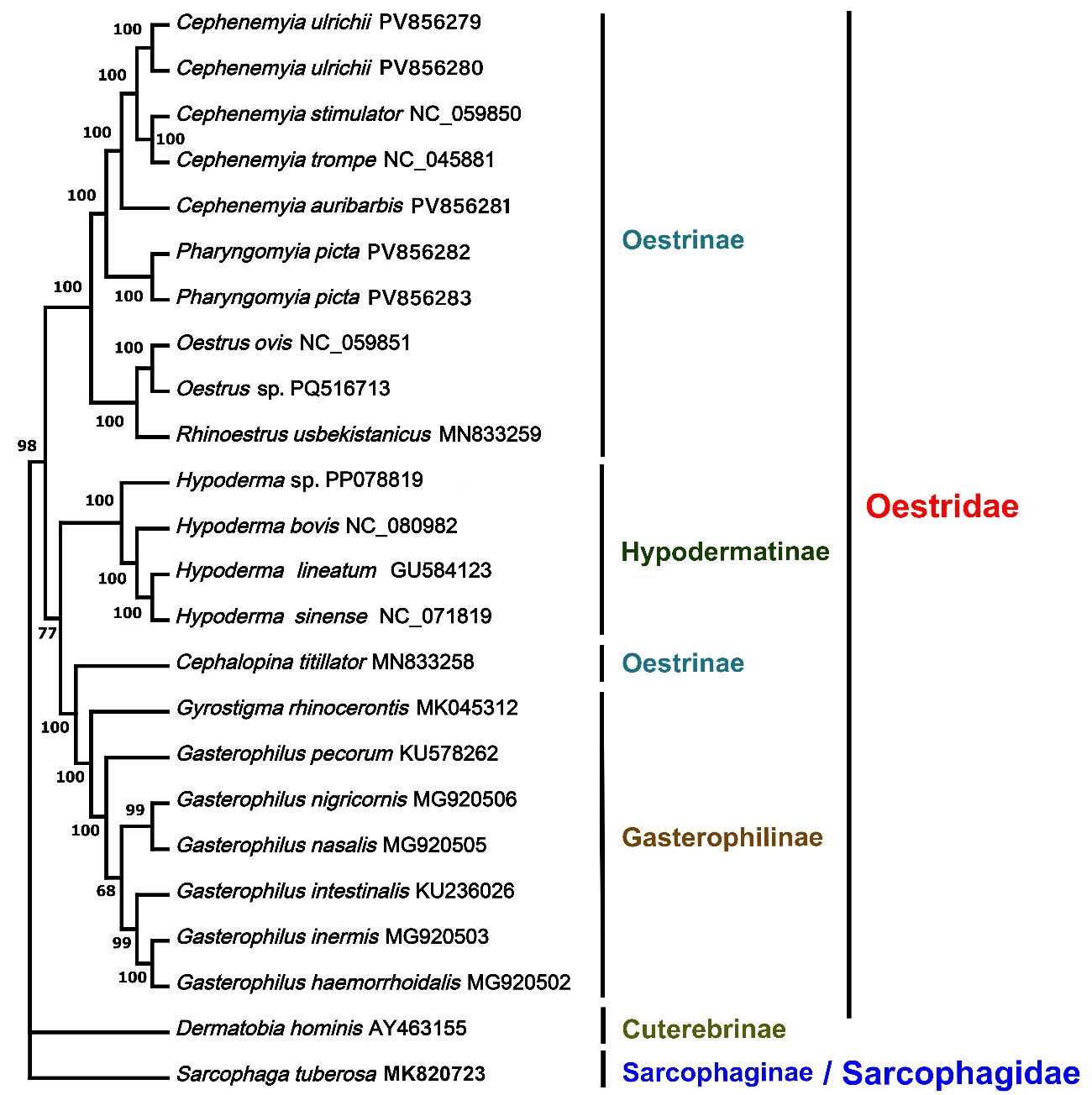

Supplement: Supplementary file 1 — Supplementary Material 1 [file 436_2025_8623_MOESM1_ESM.docx]
